# Identification of unique cardiolipin and monolysocardiolipin species in *Acinetobacter baumannii*

**DOI:** 10.1038/s41598-017-03214-w

**Published:** 2017-06-07

**Authors:** Patrizia Lopalco, Julia Stahl, Cosimo Annese, Beate Averhoff, Angela Corcelli

**Affiliations:** 1 Department of Basic Medical Sciences, Neuroscience and Sense Organs, University of Bari Aldo Moro, Bari, Italy; 2 Department of Molecular Microbiology & Bioenergetics, Institute of Molecular Biosciences, Goethe-University Frankfurt am Main, Frankfurt, Germany; 3 Italian National Council for Research - Institute for the Chemistry of OrganoMetallic Compounds (CNR-ICCOM), Bari, Italy; 4 Italian National Council for Research - Institute for Chemical-Physical Processes (CNR- IPCF), Bari, Italy

## Abstract

Acidic glycerophospholipids play an important role in determining the resistance of Gram-negative bacteria to stress conditions and antibiotics. *Acinetobacter baumannii*, an opportunistic human pathogen which is responsible for an increasing number of nosocomial infections, exhibits broad antibiotic resistances. Here lipids of *A*. *baumannii* have been analyzed by combined MALDI-TOF/MS and TLC analyses; in addition GC-MS analyses of fatty acid methyl esters released by methanolysis of membrane phospholipids have been performed. The main glycerophospholipids are phosphatidylethanolamine, phosphatidylglycerol, acyl-phosphatidylglycerol and cardiolipin together with monolysocardiolipin, a lysophospholipid only rarely detected in bacterial membranes. The major acyl chains in the phospholipids are C16:0 and C18:1, plus minor amounts of short chain fatty acids. The structures of the cardiolipin and monolysocardiolipin have been elucidated by post source decay mass spectrometry analysis. A large variety of cardiolipin and monolysocardiolipin species were found in *A*. *baumannii*. Similar lysocardiolipin levels were found in the two clinical strains *A*. *baumannii* ATCC19606﻿^T﻿^ and AYE whereas in the nonpathogenic strain *Acinetobacter baylyi* ADP1 lysocardiolipin levels were highly reduced.

## Introduction

*Acinetobacter baumannii* is a well-adapted hospital pathogen responsible for an increasing number of intensive care unit (ICU)-acquired pneumonia cases, as well as wound, tissue and urinary tract infections^1–3^. In addition to the pathogenic potential, *A*. *baumannii* epidemic lineages have developed increasing multidrug resistances and even pan-drug resistance^4, 5^. Its success as an emerging pathogen is furthermore promoted by its remarkable ability to persist on dry surfaces for weeks or even for month^6^. Survival in the human host, resistance to antibiotics and desiccation stress resistance are promoted by distinct features of the cell surface^7, 8^.

In Gram-negative bacteria the outer membrane, consisting of a monolayer of glycerophospholipids and an exposed monolayer of lipopolysaccharides (LPS) serves as the outermost barrier restricting the transfer of toxic compounds and maintaining a hydrated layer around the cell thereby enhancing resistance to different environmental stresses. Modulation of the LPS structure significantly contributes to escape immune surveillance and gain protection against host defense mechanisms; the bioactive component of LPS is lipid A^9, 10^.

The lipid A saccharolipid moiety is the most important immunostimulator center of the LPS and it is able to activate the innate immune system^11, 12^. Lipid A alterations directly affect pathogenesis, by modifying the outer membrane permeability barrier and promoting resistance to antibiotics and desiccation^12, 13^.

Recent studies on mechanisms of lipid A remodeling, which occurs through modulation of acylation processes and chain modifications, suggest an involvement of surrounding glycerophospholipids, yet to be defined in full molecular details^14, 15^.

In the Gram-negative bacterium *Salmonella thiphimurium*, glycerophospholipids cooperate with lipid A to form a critical barrier for antibiotic resistance and intracellular survival in the host. A specific role for the tetracylated phospholipid cardiolipin (CL) and the triacylated acyl-phosphatidylglycerol (Acyl-PG) has been suggested in the remodeling process of *Salmonella* lipid A^14^; it has been reported that in this organism the PhoPQ virulence regulators not only modulates the lipid A structure but also the proportions of some acidic glycerolipids in the outer membrane^16^. In *E*. *coli* the mechanism of cardiolipin trafficking from inner to outer membrane under control of the PhoPQ regulator system has been recently investigated^17^.

Glycerophospholipids of *A*. *baumannii* have been studied in the past by means of classical analytical approaches^18–24^. One of the most intriguing feature of the lipid composition of this organism is the presence of relatively high levels of monolysocardiolipin, a triacylated phospholipid rarely found in bacterial or eukaryotic membranes^23, 24^. The presence of monolysocardiolipin as a normal phospholipid component of *Acinetobacter* sp. HO1-N suggested the presence of an active phospholipase A (PLA). Indeed a PLA activity was detected in outer membrane fractions of *Acinetobacter* HO1-N, hydrolyzing cardiolipin to monolyso- and dilysocardiolipin but also hydrolyzing phosphatidylethanolamine (PE) and phosphatidylglycerol (PG)^23^. In addition another triacylated phospholipid, Acyl-PG, was previously characterized in *Acinetobacter* membranes and considered a product of intermolecular transacylation of PG by PLA activity^24^.

In recent microbiological studies the ESI-MS lipid profiles of different strains of *Acinetobacter baumannii* have been reported, however not all the classes of membrane lipids have been considered^15^.

In the present study, for the first time we analyzed lipids of *A*. *baumannii* by MALDI-TOF/MS analyses, a technique allowing simultaneous detection of many different lipid species; in addition thin layer chromatography analyses have been used to separate main lipid classes from the lipid extract of the whole organisms to gain further details on minor lipid components too. Furthermore GC-MS analyses have been performed to identify fatty acids released from phospholipids after acid hydrolysis.

We report novel findings on membrane glycerophospholipids with special focus on cardiolipins and its lysocompound monolysocardiolipin, a complex glycerophospholipid only rarely detected in bacterial and eukaryal membranes. High lysocardiolipin levels were found in the two clinical strains *A*. *baumannii* ATCC19606^T^ and AYE whereas only low levels were detected in the nonpathogenic strain *Acinetobacter baylyi* ADP1. The monolysocardiolipin level of *Acinetobacter* strains could represent a distinct lipid phenotype reflecting different roles of the glycerophospholipids in the adaptation to the environment.

## Results

### Total lipid composition of A. baumannii ATCC 19606^T^ and fatty acid analyses

**Figure 1:**
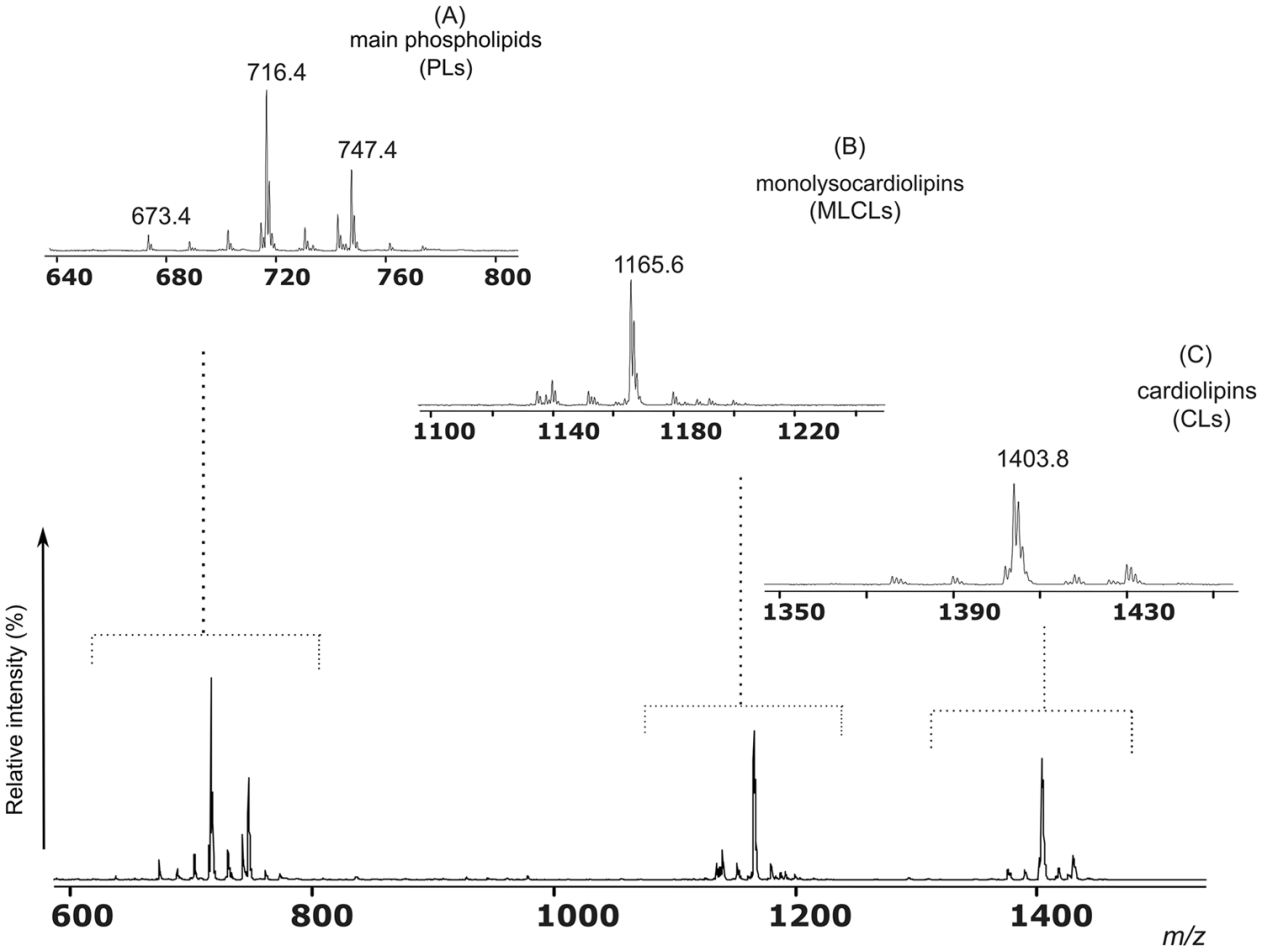
MALDI-TOF/MS analysis of the total lipid extract of *A*. *baumannii* cells. Mass spectra were acquired in negative ion mode using 9-aminoacridine as matrix. Mass spectrum in the lower panel represents the MALDI-TOF/MS lipid profile in the full *m/z* range 600–1500. In panel (**A**) x-axis enlargement of the *m/z* range 640–800 referable to the main phospholipids (PLs); in panel (**B**) x-axis enlargement of the *m/z* range 1100–1200 referable to the monolysocardiolipins (MLCLs); in panel (**C**) x-axis enlargement of the *m/z* range 1350–1450 referable to the cardiolipins (CLs). A detailed list of detected peaks is shown in Table 1 (PLs), and Table 2 (CLs, MLCLs).

In order to obtain detailed information on the lipid composition of *A*. *baumannii* ATCC 19606^T^, cells were grown in LB medium to the stationary phase, harvested and subjected to lipid extraction and the total lipid extract of *A*. *baumannii* was analyzed by MALDI-TOF/MS in the negative ion mode (Fig. 1). The peaks in the MALDI-TOF/MS lipid profile can be grouped in three main *m/z* ranges: panel A shows the interval *m/z* 640–800 where the peaks of main phospholipids (PLs) are present; panel B shows an enlargement of the *m/z* interval 1100–1200 containing the monolysocardiolipins (MLCLs) peaks; and panel C shows the cardiolipins (CLs) in the *m/z* range 1350–1450 (Fig. 1). The major phospholipid signals are at *m/*z 716.4 and 747.4, attributable to phosphatidylethanolamine (PE) (34:1) and phosphatidylglycerol (PG) (34:1) respectively; in addition, minor peaks attributable to other species of PE and PG are also present.

In the *m/z* ranges of MLCLs and CLs the main peaks are at *m/z* 1165.6 attributable to MLCL (52:2) and *m/z* 1403.8 attributable to CL (68:2).

Major and minor peaks corresponding to the lipid species present in the total lipid extract are listed in Tables 1 and 2, the latter specifically dedicated to CL and MLCL species.

**Table 1:** Lipid assignments of *m/z* values in MALDI –TOF mass spectra (negative ion mode) of the total lipid extract of *Acinetobacter baumannii*.

Phospholipid	*m/z*	[M-H]^-^	Assignment
LPE	478.272	478.301	18:1
unknown	637.295	—	—
	653.265	—	—
PG	691.129	691.463	30:1
	719.373	719.494	32:1
	733.408	733.510	33:1
	735.357	735.525	33:0
	745.424	745.510	34:2
	747.434	747.525	34:1
PA	673.380	673.489	34:1
PE	606.293	606.421	26:0
	688.396	688.500	32:1
	702.413	702.515	33:1
	714.418	714.515	34:2
	716.437	716.531	34:1
	742.446	742.546	36:2
PE-OH	622.291	622.416	26:0
	730.438	730.510	34:2
Acyl-PG	875.424	875.646	42:0
	985.625	985.755	50:1
	1011.642	1011.771	52:2

The numbers (x:y) denote the total length (as carbon numbers) and number of double bonds of both acyl chains respectively.

**Table 2:** Lipid assignments of *m/z* values in MALDI –TOF (−) spectra of the cardiolipins and monolysocardiolipins of the total lipid extract of *Acinetobacter baumannii*.

Cardiolipins	*m/z*	[M-H]^−^	Assignment
CL	1183.594	1183.784	52:0
	1293.712	1293.894	60:1
	1309.693	1309.925	61:0
	1375.776	1375.972	66:2
	1389.801	1389.988	67:2
	1403.851	1404.003	68:2
	1417.927	1418.019	69:2
	1429.848	1430.019	70:3
CL-OH	1199.573	1199.779	52:0
**Monolysocardiolipins**			
MLCL	945.388	945.555	36:0
	1029.503	1029.649	42:0
	1055.521	1055.664	44:1
	1071.519	1071.696	45:0
	1137.612	1137.743	50:2
	1139.616	1139.758	50:1
	1165.657	1165.774	52:2
MLCL-OH	1045.499	1045.644	42:0

The numbers (x:y) denote the total length (as carbon numbers) and number of double bonds of both acyl chains respectively.

To support the mass spectrometry peak assignements, fatty acid analyses have been performed.

**Figure 2:**
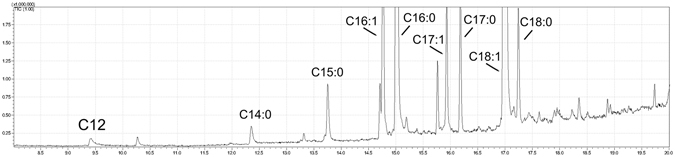
GC-MS analyses of FAMEs released by acid methanolysis from the total lipid extract. Carbon atoms and unsaturations of various fatty acid chains are reported.

Figure 2 shows the chromatogram of FAMEs released by acidic methanolysis from the total lipid extract of *Acinetobacter baumannii*. Main fatty acids are C16:0 and C18:1; C16:1 and C18:0 are also present plus minor amounts of C17:0, C17:1, C15:0, C14:0 and C12:0 in order of abundance.

**Figure 3:**
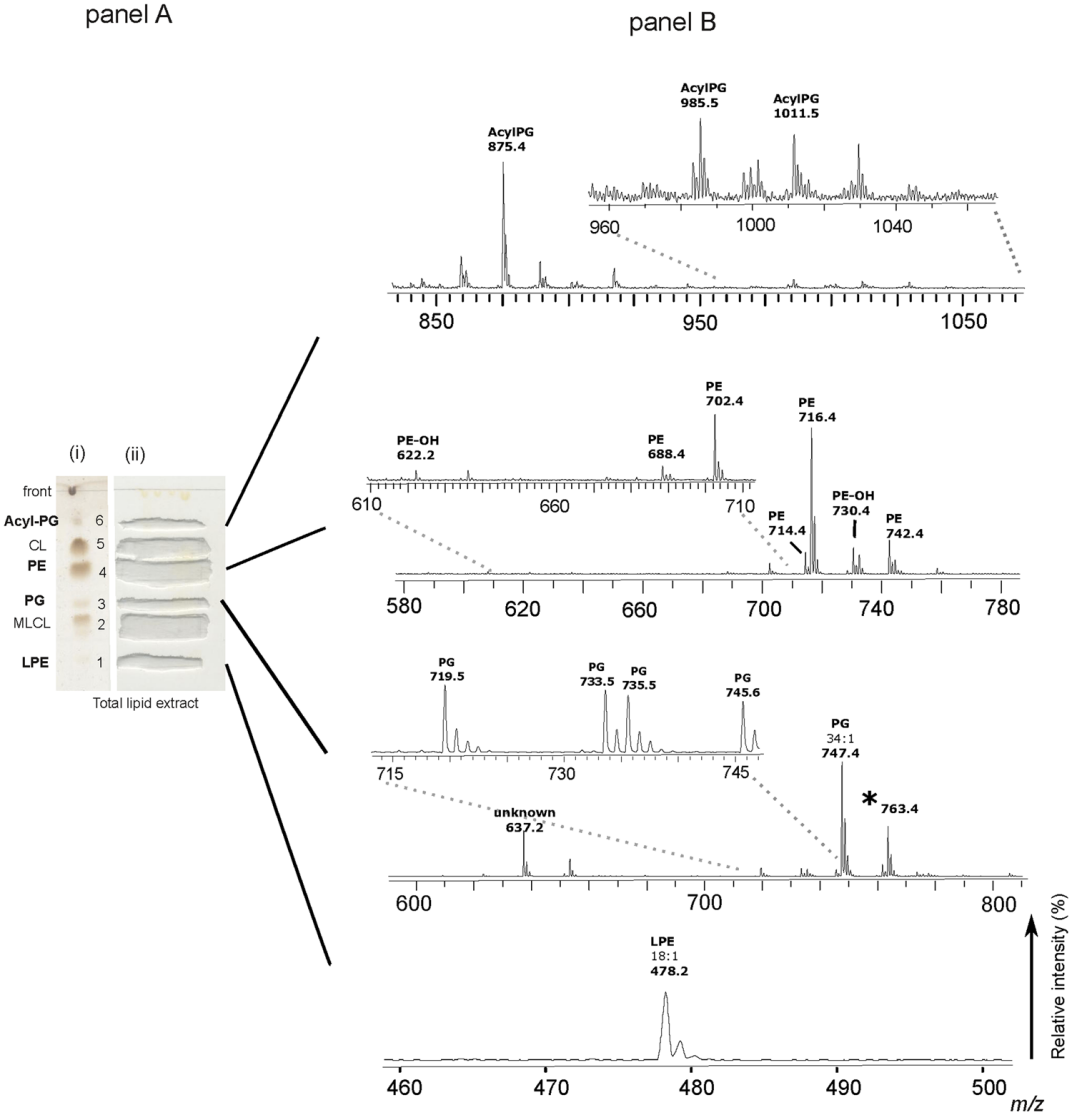
MALDI-TOF/MS analyses of lipid bands #1, 3, 4 and 6, isolated from the total lipid extract of *A*. *baumannii* by TLC. The total lipid extract of *A*. *baumannii* cells was loaded on the plate (160 μg per each lane). One lane was sprayed with sulfuric acid and charred in the oven (permanent staining of all class of lipids) (i); the other lanes were stained with iodine vapors (temporary staining of all class of lipids) (ii); each band was marked with a pencil and silica was scraped in correspondence of lipid bands. The chromatography plate is shown in panel A. Lipid bands #1, 3, 4 and 6 were extracted from silica and were analyzed by MALDI-TOF/MS. The MALDI/TOF-MS (negative ion mode) spectra of the four lipid bands corresponding to Acyl-PGs, PE, PG and LPE (from the top to the bottom) are shown in panel B. The detailed list of detected peaks is shown in Table 1. The peak at *m/z* 763.4 labeled with star has not been included in the list because it results from TLC artifact.

**Figure 4:**
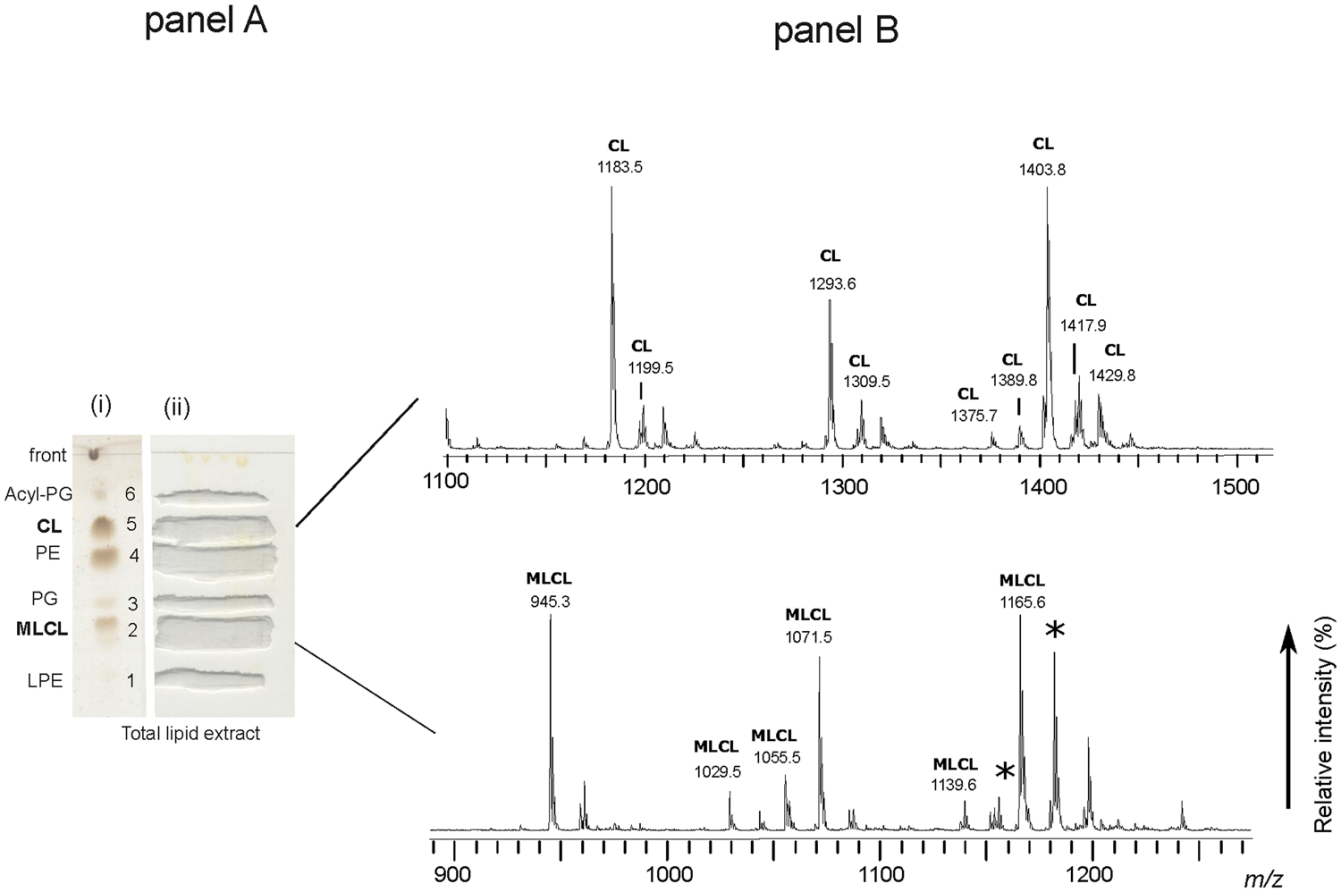
MALDI-TOF/MS analyses of lipid bands #2 and 5, isolated from the total lipid extract of *A*. *baumannii* by TLC. The chromatography plate is shown in panel A. Lipid bands #2 and 5 were extracted from silica and were analyzed by MALDI-TOF/MS. The MALDI/TOF-MS (negative ion mode) spectra of the two lipid bands corresponding to CLs (top), MLCLs (bottom) are shown in panel B. The detailed list of detected peaks is shown in Table 2. Peaks labeled with star have been attributed to TLC artifacts.

Then the components of the lipid extract were isolated by preparative TLC (see panel A in Figs 3 and 4). Six bands of polar lipids stained with iodine vapours were scraped from the plate; then the lipids were extracted from silica by using a modified Bligh and Dyer method. All TLC bands were analyzed by MALDI-TOF/MS. Interestingly, below the PG band, a broad band attributable to MLCL is present.

### Identification of PG and Acyl-PG, PE and LPE species

MALDI-TOF/MS analyses of the bands #1, 3, 4 and 6 corresponding to lysoPE (LPE), PG, PE and Acyl-PG are shown in panel B of Fig. 3.

The main species in band #3 is that corresponding to PG 34:1 at *m/z* 747.4. The peak at 763.4 corresponds to the hydroxylated PG 34:1 (747 + 16); its structure has been elucidated by means of post source decay (PSD) analyses (data not shown); however, as it is missing in the total lipid extract, the peak at *m/z* 763.4 has to be considered the results of nonenzymatic oxidation of PG 34:1 during the isolation procedure. Four other minor PGs have been identified in band #3, all clearly visible by zooming in the MS lipid profile of total lipid extract (not shown) and all listed in Table 1. In addition a peak at *m/z* 637.2 is present in band #3; this peak has been analysed by PSD analyses, obtaining a fragmentation pattern not fitting with the standard fragmentation pattern of PG; the peak at *m/z* 637.2 is also clearly visible by zooming in the MS lipid profile of total lipid extract but its nature remains unknown (not shown).

The main acyl-PG species in band #6 (Fig. 3 panel B) is that corresponding to the peak at *m/z* 875.4 that could correspond to a triacylated phospholipid carrying short fatty acid chains; minor acyl-PGs previously described in *Salmonella thyphimurium* outer membrane^14^ are at *m/z* 985.5 and 1011.5.

The main PE species at *m/z* 716.4 (band #4) is constituted by oleic and palmitic acid chains (PE 34:1), according to also GC-MS analyses of FAME released by the PE isolated band (not shown). Furthermore the two minor peaks at *m/z* 714.4 and 742.4 have been attributed to PE (34:2) and PE (36:2) respectively; PE species carrying short fatty acid chains have been attributed to the peaks at 688.4 and 702.4. Finally the peaks at *m/z* 622.2 and 730.4 could correspond to hydroxylated PE (26:0) and (34:2) respectively. Above PE species (all listed in Table 1) could be recognized by zooming in the profile of the total lipid extract.

Lysophosphatidylethanolamine was recognized in the band of highest retention factor on TLC (band #1); MALDI-TOF/MS analyses revealed a peak at *m/z* 478.2 compatible with a lysocompound carrying an oleic acid chain (Fig. 3, panel B bottom). In addition, in the LPE band (#1) preliminary evidence for the presence of minor amounts of dilysocardiolipin has been also obtained (data not shown).

Altogether the analyses of individual TLC bands confirmed the presence of the glycerophospholipids previously identified in the total lipid extracts and displayed the presence of minor lipid components such as the triacylated phospholipid Acyl-PG and the lyso- compound LPE. The major fatty acids in the above glycerophospholipids are palmitic and oleic together with minor amounts of short chains having either even or odd number of carbon atoms.

### Cardiolipin and monolysocardiolipin fingerprintings

MALDI-TOF/MS analyses of the MLCL and CL bands (corresponding to bands #2 and 5, respectively) are reported in panel B of Fig. 4.

**Figure 5:**
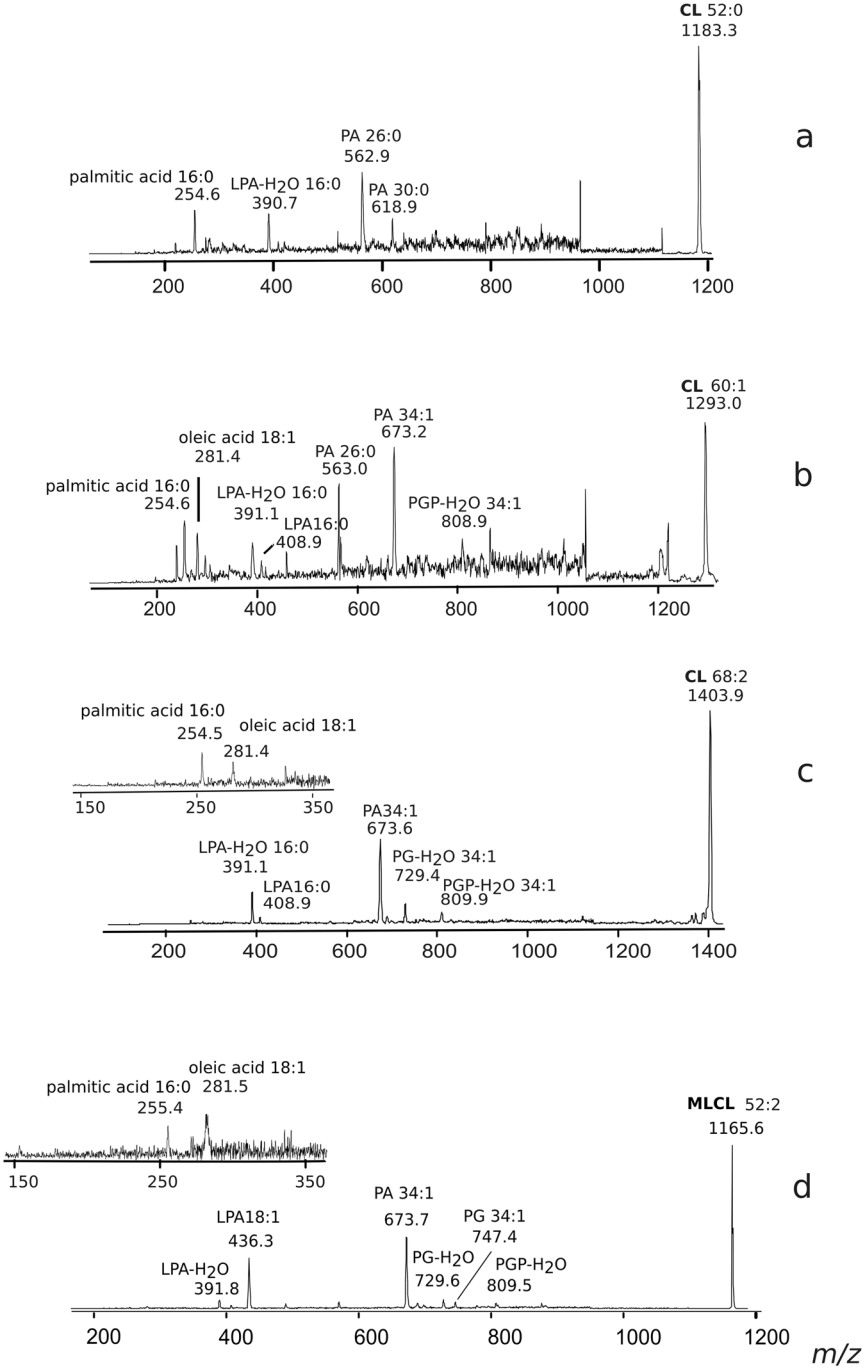
Fragmentation patterns of monolysocardiolipin and cardiolipins. PSD analysis of the peaks at *m/z* 1183.3 (**a**) 1293.0 (**b**), 1403.9 (**c**) and 1165.6 (**d**). (**a**) PSD analysis of the peak at *m/z* 1183.3: ion fragments correspond to PA (*m/z* 618.9), PA (*m/z* 562.9), LPA-H_2_O (*m/z* 390.7) and the fatty acid 16:0 (*m/z* 254.6). (**b**) PSD analysis of the peak at *m/z* 1293.0: ion fragments correspond to PGP-H_2_O (*m/z* 808.9), PA (*m/z* 673.2), PA (*m/z* 563.0), LPA (*m/z* 408.9), LPA-H_2_O (*m/z* 391.1) and fatty acids 18:1 (*m/z* 281.4) and 16:0 (*m/z* 254.6). (**c**) PSD analysis of the peak at *m/z* 1403.9: ion fragments correspond to PGP-H_2_O (*m/z* 809.9), PG-H_2_O (*m/z* 729.4), PA (*m/z* 673.6), LPA (*m/z* 408.9), LPA-H_2_O (*m/z* 391.1). In the x-axis enlargement of *m/z* range 150–350, two peaks are referable to the fatty acids 18:1 (*m/z* 281.4) and 16:0 (*m/z* 254.5). (**d**) PSD analysis of the peak at *m/z* 1165.6: ion fragments correspond to PGP-H_2_O (*m/z* 809.5), PG (*m/z* 747.4), PGP-H_2_O (*m/z* 729.6), PA (*m/z* 673.7), LPA (*m/z* 436.3), LPA-H_2_O (*m/z* 391.8). In the x-axis enlargement of *m/z* range 150–350, two peaks are referable to fatty acids 18:1 (*m/z* 281.5) and 16:0 (*m/z* 255.4).

In the cardiolipin band #5, three main peaks at *m/z* 1183.5, 1293.6 and 1403.8, together with a number of minor species, are present. PSD analyses of the three main cardiolipin peaks are shown in Fig. 5. By comparatively analysing the fragmentation patterns, we propose that the peak at *m/z* 1183.5 and 1293.6 correspond to a cardiolipin species carrying short fatty acid chains likely C12:0 and/or C14:0 in agreement with above GC-MS analyses. Minor peaks are at *m/z* 1199.5, 1309.5, 1375.7, 1389.8, 1417.9 and 1429.8 whose assignments are shown in Table 2. Among these CLs, there are species likely carrying odd carbon atom fatty acids (C15:0, C17:0 and C17:1) with the exception of the CL corresponding to the peak at *m/z* 1199.5 which has been attributed to an hydroxylated cardiolipin.

The main peaks in band #2 are at *m/z* 945.3, 1071.5 and 1165.6 corresponding to MLCL species. In the PSD fragmentation pattern of the molecular ion at *m/z* 1165.6, peaks at *m/z* 281.5, 391.8, 673.7, 747.4 and 809.5 were present (see panel D in Fig. 5).

The peaks at *m/z* 945.3, 1029.5, 1045.4, 1055.5 and 1071.5 have been assigned to MLCLs carrying short fatty acid chains. The peaks at *m/z* 1155.4 and 1181.4 labeled with the star are artifacts due to the TLC, as they are not present in the lipid extract before chromatography.

**Figure 6:**
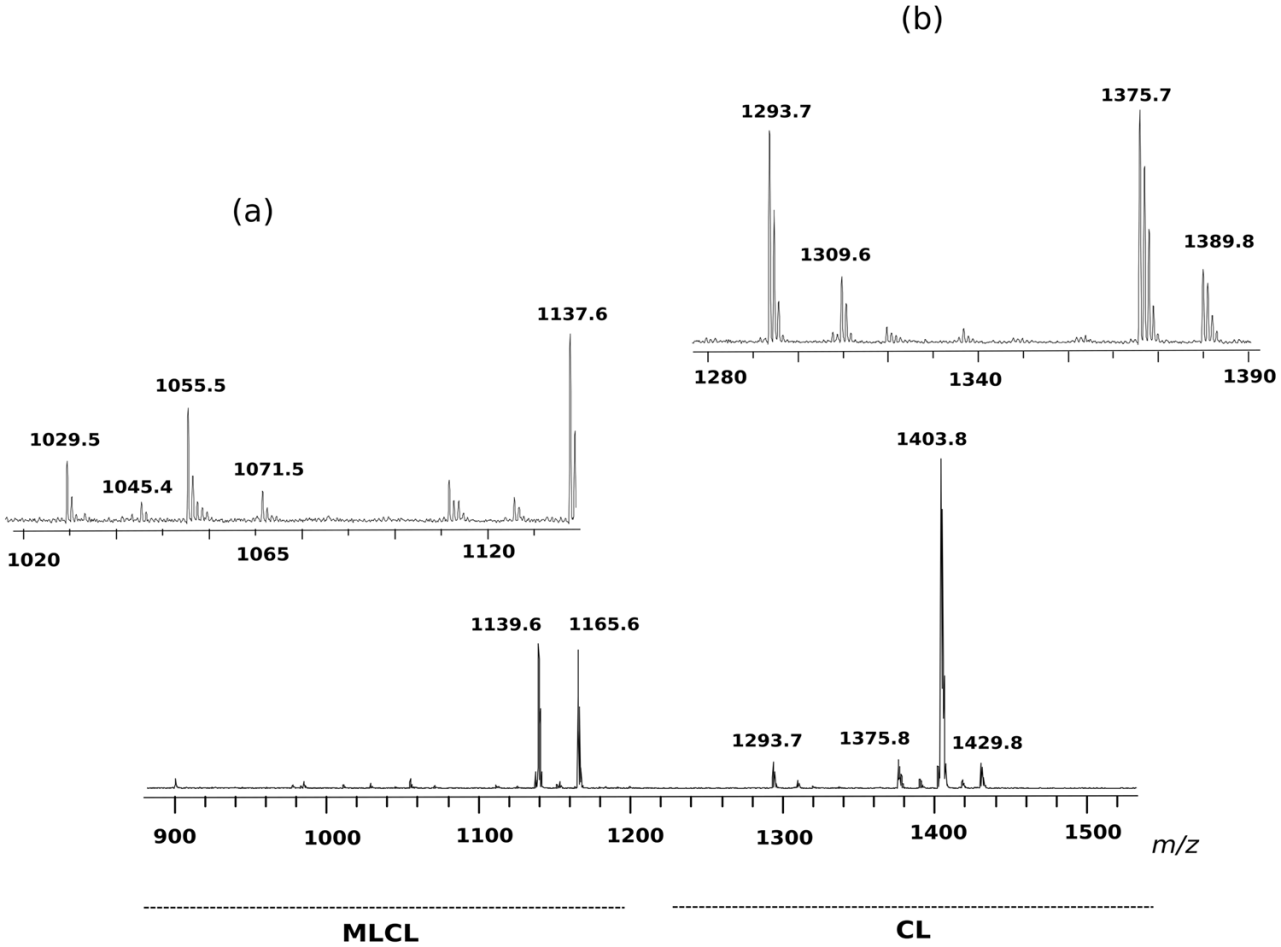
MLCL and CL fingerprint of intact membranes of *Acinetobacter baumannii*, acquired by MALDI-TOF/MS analyses. (**a**) *m/z* range of MLCLs; (**b**) *m/z* range of CLs.

Further investigations on MLCL and CL species have been carried out by using an innovative experimental approach in which membrane lipids of the organism have been analysed in intact membranes by completely avoiding lipid extraction and TLC separation steps, by following a procedure previously described in the literature that highly reduces the times of analyses and the possibility of introducing artifacts^25^. The full lipid profile of intact membranes is shown in Fig. S1 of supplementary information, all the molecular species in the lipid extract were also present in the lipid profile of intact membranes. Zooms in the *m/z* range of cardiolipin and monolysocardiolipin shown in Fig. 6 confirm the presence of MLCL and CL species previously described; the differences in the relative peak heights likely depend on the different conditions of ionization. The main advantage of the present method over other approaches is that it can simultaneously detect CL and MLCL species in the total lipid profile by a single run of mass spectrometric analysis; with no doubt, the success of this experimental approach is due to the use of 9-AA as matrix, which is particularly suitable for CLs and MLCLs detection even when these species are only minor components in complex lipid profiles^25^.

Therefore we conclude that the large monolysocardiolipin band contains short and long chain monolysocardiolipin species. The presence of relatively high levels of MLCL in the lipid profile of *A*. *baumannii* represents an interesting feature, almost unique in bacteria and rarely seen in mitochondria where, in general, the appearance of MLCL is associated with impairment of bioenergetic functions^26^.

In summary we have shown that cardiolipins and monolysocardiolipins are constituted by a particular combination of C16:0, C18:1 and minor amounts of the short chain fatty acids C12:0 and C14:0. Molecular diversity is generated by the combinatorial properties of different acyl substitutions, which for tetra-acylated molecules like CL, can be quite large as previously considered^27, 28^.

### Pathogenic A. baumannii ATCC 19606^T^ and AYE strains exhibit higher MLCL levels than non-pathogenic Acinetobacter baylyi

**Figure 7:**
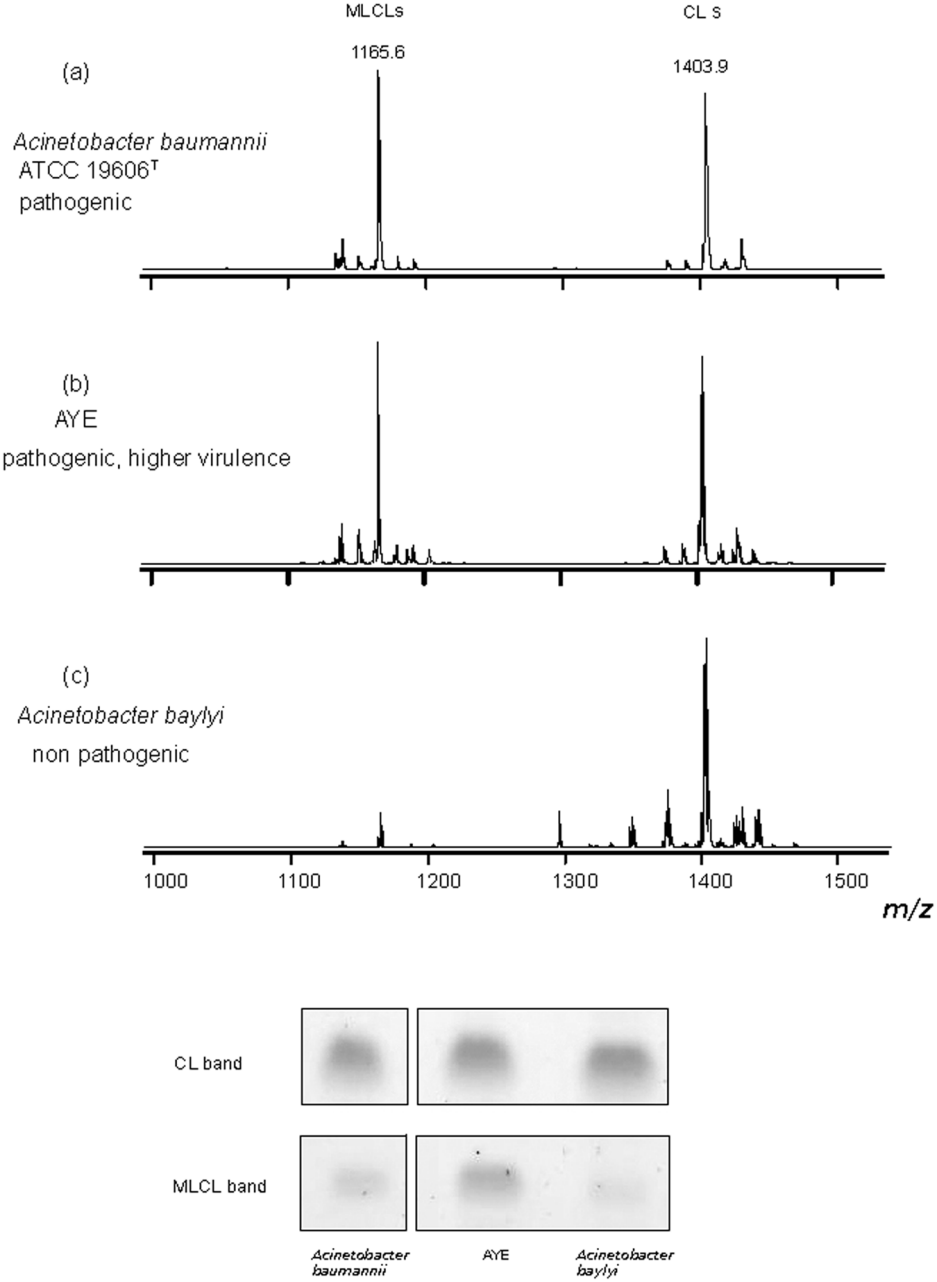
MLCL and CL levels of *A*. *baumannii*, AYE *and A*. *baylyi*. The lipid analyses of high and low virulence cells have been performed by combining mass spectrometry and TLC. Mass spectra were acquired in negative ion mode using 9-aminoacridine as matrix. The *m/z* range 1000–1500 refers to monolysocardiolipins (MLCLs) and cardiolipins (CLs). At the bottom the TLC bands of CL and MLCL in the three strains.

To get insights into the distribution of MLCLs and CLs in other *Acinetobacter* strains the lipid profiles of a multi resistant member of the clonal lineage I *A*. *baumannii*, AYE and the non-pathogenic soil bacterium *A*. *baylyi* were analyzed by mass spectrometry and thin layer chromatography. The Fig. 7 illustrates the MLCL and CL zooms of the MALDI-TOF/MS lipid profiles and the CL and MLCL bands on TLC of the different strains considered in the present study; it can be seen that the amount of CLs is comparable in the three strains, while the amount of MLCLs is much lower in the non pathogenic strain *A*. *baylyi*. This shows that MLCL levels differ in *Acinetobacter* strains. Different levels of MLCLs could be the result of different expression levels and/or activity of the bacterial transacylases playing a role in the mechanism of lipid A remodeling.

## Discussion

Glycerophospholipids represent important membrane constituents of Gram-negative bacteria. Besides the structural role, glycerophospholipids support membrane proteins in their specific functions such as transport across membranes, bioenergetic processes and quorum sensing. Membrane functions depend on lipid-protein interactions that can occur through specific interaction of the phospholipid polar heads with amino acid charged residues and/or very specific interactions of lipid chains with proteins as demonstrated by X-ray studies^29^ and recent elegant NMR studies^30^.

Most common bacterial glycerophospholipids contain two fatty acid chains. In addition minor amounts of acylated phosphodiglycerides containing three fatty acid chains, such as Acyl-PG and acyl-phosphatidylethanolamine, have been found in several Gram-negative bacteria^23, 31, 32^. As regards the dimeric phospholipid cardiolipin, which is constituted by four fatty acid chains, it is known that it can be present in different proportions in bacterial membranes, preferentially accumulated at the cell poles in rod-shaped bacteria and that its levels can be modulated by environmental factors such as oxygen availability and osmotic stress^33–37^. Cardiolipin has been found to be invariably associated to a number of prokaryotic respiratory complexes^29, 33, 38–40^. Moreover, it is clear that polar and septal accumulation of cardiolipin in a number of prokaryotes may ensure proper spatial segregation and/or activity of proteins and recruitment of proteins to the poles^36, 37, 41^. For example proper functioning of the glycine-betaine transporter proP of *Escherichia coli* was shown to be dependent on cardiolipin present at the cell poles. Therefore a distinct role of the cardiolipin content of the *E*. *coli* membranes was proposed for osmo-adaptation of this bacterium^39, 42^.

Glycerophospholipids contribute to the formation of the lipid bilayer or matrix, while the non bilayer cone shaped phospholipids exert different roles in tight association with membrane proteins.

Phosphatidic acid and cardiolipin, which represent anionic non bilayer phospholipids, are considered to be important membrane constituents conferring peculiar permeability properties to the outer bacterial membranes and specifically contributing to antibiotic resistance^43, 44^. The triacylated glycerophospholipids, Acyl-PG and monolysocardiolipin, can also be considered non-bilayer lipids and their presence might also contribute in fortifying the membrane of Gram-negative bacteria against certain antibiotics.

The data of the present study expand the knowledge on the glycerophospholipids present in the lipid extract of *A*. *baumannii*. Detailed information on lipid classes, their proportion in membrane and chain composition of lipids have been obtained by integrating data collected by means of different analytical approaches, including MALDI-TOF/MS analyses of intact membranes of *Acinetobacter baumannii*. The lipid analysis of intact membranes allows to skip lipid extraction and separation steps, highly reducing the time required for the analyses and the possibility to introduce artifacts. Only minute amounts of sample are required to perform lipid analyses in intact mode, thus introducing the possibility to quickly screen mutants and, most importantly, specific membrane domains of the bacterial membranes of the organism under study.

Thin layer chromatography analyses have shown that PE is an important component in the lipid extract together with cardiolipin and its lyso-compound monolysocardiolipin. In addition the presence of various species of PG, Acyl-PG and LPE as minor lipid components, has also been documented by our lipidomic approach. GC-MS analyses showed that together with C16 and C18 fatty acid chains, short C12 and C14 chains are present in the phospholipids. Some evidence for chain hydroxylation in PE, CL and MLCL has also been obtained in our study.

Noteworthy, recently it has been reported that polymyxin resistant *A. baumannii* has higher amounts of short fatty acid chains in glycerophospholipids compared to polymyxin sensitive strains^45^.

The presence of four distinct acyl positions in cardiolipin allows the formation of a large number of molecular species, by different combinations of n acyl groups; based on combinatorial consideratons, n^4^ different molecular species can be formed. The main fatty acids in the CL are palmitic and oleic acids; one or two short chains can also be present. With only three different acyl groups randomly distributed on the four different positions, 3^4^ (81) cardiolipin molecules can be potentially formed; if the number of available acyl groups is doubled combining hydroxylated or not fatty acid chains, then the number of different CL molecules is huge 6^4^ (1296). The molecular diversity of CL can be considered an important resource for the remodeling processes of the bacterial membranes.

Our working hypothesis for future studies is that CL and MLCL containing short chains might be involved in lipid A remodeling, thereby affecting virulence of *A*. *baumannii*. It is known that in *A*. *baumannii* the mechanism of lipid A (hepta-)acylation is independent of the PagP enzyme system, since no genes encoding for such a cluster are encoded in the *A*. *baumannii* genome. Therefore lipid A acylation of *A*. *baumannii* is different from that of *Escherichia coli* and *Salmonella*. In *A*. *baumannii* LpxL_Ab_ and LpxM_Ab_ acyl transferases are responsible for the transfer of short fatty acid chains during lipid A biosynthesis^13^. Membrane phospholipid remodeling in Gram-negative bacteria by lipid transfer and acylation processes has been recently investigated^17, 46^. The coexistence of a variety of MLCLs and CLs in the membranes of *A*. *baumannii* supports the idea of a dynamic CL remodeling possibly associated with the lipid A remodeling as specific response to environmental and quorum sensing activity changes.

Further analytical studies elucidating the specific lipid composition of the inner and outer membrane, together with the study of outer membrane biogenesis and the regulatory systems that sense and respond to stress, will shed light onto the possible role of CL and MLCL in lipid A remodeling.

## Materials and Methods

### Materials

9-Aminoacridine hemihydrate (9-AA) was purchased from Acros Organics (Morris Plains, NJ). All organic solvents used in extraction and MS analyses were commercially distilled, of the highest available purity, and purchased from Sigma-Aldrich, J. T. Baker, or Carlo Erba. The following commercial glycerophospholipids (used as standards) 1,2-dimyristoyl-*sn*-glycero-3-phosphate, 1,2-dimyristoyl-*sn*-glycero-3-phospho-(1′-rac-glycerol), 1,2-dimyristoyl-*sn*-glycero-3-phospho-L-serine, 1,2-diphytanoyl-*sn*-glycero-3-phosphoethanolamine, 1′,3′-bis[1,2-dimyristoyl-*sn*-glycero-3-phospho]-*sn*-glycerol, 1′,3′-bis[1,2-dioleoyl-*sn*-glycero-3-phospho]-*sn*-glycerol, were purchased from Avanti Polar Lipids (Alabaster, AL).

### Bacterial strain and culture conditions

*Acinetobacter baumannii* strain ATCC 19606^T^ and AYE were grown in complex medium^47^ at 37 °C whereas *Acinetobacter baylyi* ADP1 was grown at 30 °C. Cells were harvested in the stationary phase, washed twice in 0.9% NaCl, frozen in liquid nitrogen and stored at −60 °C until use for lipid extraction.

### Lipid extraction

Total lipids were extracted from frozen and thawed cells using the Bligh and Dyer method (methanol/chloroform/water; 2:1:0.8, by volume)^48^. The extracts were dried under N_2_ before weighing and then dissolved in chloroform (final concentration 10 mg/ml).

### GC-MS analyses

Samples were prepared as previously described^48^; briefly, a chloroform solution of the total lipid extract (about 1 mg) was dried in a stream of nitrogen at 30 °C, and then refluxed in 0.5 ml methanolic-hydrochloric acid (0.6 N) for 3 h at 80 °C. Fatty acid methyl esters were extracted, in hexane. The hexane extracts were dried under N_2_ and then dissolved in chloroform. Fatty acid methyl ester (FAME) composition was determined by gas chromatography with mass spectrometry (GC-MS).

The GC–MS analyses were carried out on a Shimadzu GC 2010 plus gas chromatograph coupled to a Shimadzu GC-MS QP2010 SE mass spectrometer. The chromatographic separations were performed using a SLB-5ms column (30 m × 0.25 mm id, film thickness 0.25 μm). The GC parameters were the following: gas carrier (helium) at the constant flow rate of 0.87 ml/min; the injector (split mode) was at 250 °C; the oven temperature program was 120 °C (5 min) to 180 °C (3 min) at 20 °C/min, to 280 °C (20 min) at 10 °C/min. The MS spectra were obtained in EI mode (70 eV) with the ion source temperature at 200 °C.

### TLC analyses

Total lipid extracts were analyzed by thin layer chromatography (TLC) on silica gel 60 A plates (Merck, 20 × 10 cm, layer thickness 0.2 mm). The plates were washed twice with chloroform/methanol (1:1, by volume) and activated at 180 °C before use. Polar lipids were eluted with Solvent A (chloroform/methanol/acetic acid/water 85:15:10:3.5, by volume).

Lipid detection was carried out by spraying the plate with 5% sulfuric acid in water, followed by charring at 180 *°*C for 5 min; moreover, the following stainings were performed in order to identify the lipid classes present in the TLC bands: (i) molybdenum blue spray reagent (Sigma-Aldrich) specific for phospholipids, and (ii) ninhydrin solution, prepared dissolving 0.25 g of reagent grade ninhydrin in 100 ml of acetone-lutidine (9:1, by volume), for phosphatides or lipids having a free amino group^48^.

### Isolation and purification of individual lipids from the total extract

The lipid components of the total lipid extract of *A*. *baumannii* were separated by TLC (Merck 20 × 10 cm × 0.2 mm thick layer, glass plates) in Solvent A. Lipids were visualized by staining with iodine vapour and were eluted and recovered from the scraped silica, as previously described^48^. Isolated and purified phospholipids were dissolved in chloroform (1 mg/ml).

### Preparation of lipid extracts and intact membranes for MALDI-TOF/MS lipid analysis

For lipid samples in solution, 5 μl of the solution in chloroform were diluted in 45 μl of 2-propanol/acetonitrile (60/40, by volume), then 10 μl of the diluted solution were mixed with 10 μl of matrix solution (9-AA, 10 mg/ml in 2-propanol/acetonitrile, 60/40, by volume), as previously described^49^. The resulting lipids-matrix solution was then spotted onto the MALDI target (Micro Scout Plate, MSP 96 ground steel target) in droplets of 0.35 μl and analyzed as described below.

Whole cells of the microorganism were double washed in low salt containing medium or water before lipid analyses ﻿of intact membranes. The bacterial membrane suspensions were diluted to 1 μg/μl of total membrane protein concentration, determined by Bradford assay (Bio-Rad Protein Assay Kit; BioRad Laboratories, Germany). Then 1 μl of bacterial membrane suspension was spotted onto the MALDI target. After water evaporation a thin layer (0.35 μl) of matrix solution (9-AA, 10 mg/ml in 2-propanol/acetonitrile, 60/40, v/v) was spotted on the dried sample. After the evaporation of the matrix solvent, the samples are ready to be directly analysed with MALDI-TOF/MS.

### MALDI-TOF mass spectrometry

MALDI-TOF mass spectra of lipid extracts and intact membranes were generally acquired on a Bruker Microflex LRF mass spectrometer (Bruker Daltonics, Bremen, Germany). The system utilizes a pulsed nitrogen laser, emitting at 337 nm, the extraction voltage was 20 kV and gated matrix suppression was applied to prevent detector saturation. A total of 999 single laser shots (sum of 3 × 333) were averaged for each mass spectrum. The laser fluence was kept about 10% above threshold to have a good signal-to-noise ratio. All spectra were acquired in reflector mode using the delayed pulsed extraction; spectra acquired in negative ion mode are shown in this study. Spectral mass resolutions and signal-to-noise ratios were determined by the software for the instrment, “Flex Analysis 3.3” (Bruker Daltonics).

Post Source Decay (PSD) spectra were acquired on a Bruker Microflex mass spectrometer (Bruker Daltonics, Bremen, Germany), as previously described^50^. Briefly, the precursor ions were isolated using a time ion selector. The fragment ions were refocused onto the detector by stepping the voltage applied to the reflectron in appropriate increments. This was done automatically by using the “FAST” (fragment analysis and structural TOF) subroutine of the Flex Analysis software. Mass accuracy of our instrument is 200 ppm (external calibration).

A mix containing: 1,2-dimyristoyl-*sn*-glycero-3-phosphate, 1,2-dimyristoyl-*sn*-glycero-3-phospho-(1′-rac-glycerol), 1,2-dimyristoyl-*sn*-glycero-3-phospho-L-serine, 1,2-diphytanoyl-*sn*-glycero-3-phosphoethanolamine, 1′,3′-bis[1,2-dimyristoyl-*sn*-glycero-3-phospho]-*sn*-glycerol, 1′,3′-bis[1,2-dioleoyl-*sn*-glycero-3-phospho]-*sn*-glycerol, was always spotted next to the sample as external standard and an external calibration was performed before each measurement; the mass range of the authentic standards is 590–1450 *amu*.

## Electronic supplementary material

Figure S1

## References

[1] Peleg AY (2012). The Success of Acinetobacter Species; Genetic, Metabolic and Virulence Attributes. PLoS ONE.

[2] Dijkshoorn L, Nemec A, Seifert H (2007). An increasing threat in hospitals: multidrug-resistant *Acinetobacter baumannii*. Nat. Rev. Microbiol..

[3] Averhoff B (2015). *Acinetobacter baumannii* - understanding and fighting a new emerging pathogen. Environ. Microbiol. Rep..

[4] Perez F (2007). Global challenge of multidrug-resistant *Acinetobacter baumannii*. Antimicrob. Agents. Chemother..

[5] Peleg AY, Seifert H, Paterson DL (2008). *Acinetobacter baumannii*: emergence of a successful pathogen. Clin. Microbiol. Rev..

[6] Giannouli M (2013). Virulence-related traits of epidemic *Acinetobacter baumannii* strains belonging to the international clonal lineages I-III and to the emerging genotypes ST25 and ST78. BMC Infect. Dis..

[7] Weber BS, Harding CM, Feldman MF (2015). Pathogenic Acinetobacter: from the cell surface to infinity and beyond. J. Bacteriol..

[8] Wong D (2017). Clinical and pathophysiological overview of Acinetobacter infections: a century of challenges. Clin. Microbiol. Rev..

[9] Needham BD, Trent MS (2013). Fortifying the barrier: the impact of lipid A remodelling on bacterial pathogenesis. Nat. Rev. Microbiol..

[10] LaRock DL, Chaudhary A, Miller SI (2015). Salmonellae interactions with host processes. Nat. Rev. Microbiol..

[11] Leone S (2007). Detailed characterization of the lipid A fraction from the nonpathogen *Acinetobacter radioresistens* strain S13. J. Lipid. Res..

[12] Korneev KV (2015). Structural relationship of the lipid A acyl groups to activation of murine toll-like receptor 4 by Lipopolysaccharides from pathogenic strains of *Burkholderia mallei*, *Acinetobacter baumannii*, and *Pseudomonas aeruginosa*. Front. Immunol..

[13] Boll JM (2015). Reinforcing lipid A acylation on the cell surface of *Acinetobacter baumannii* promotes cationic antimicrobial peptide resistance and desiccation survival. mBio.

[14] Dalebroux ZD, Matamouros S, Whittington D, Bishop RE, Miller SI (2014). PhoPQ regulates acidic glycerophospholipid content of the *Salmonella Typhimurium* outer membrane. Proc. Natl. Acad. Sci. USA.

[15] Boll JM (2016). A penicillin-binding protein inhibits selection of colistin-resistant, lipooligosaccharide deficient *Acinetobacter baumannii*. Proc. Natl. Acad. Sci. USA.

[16] Dalebroux ZD (2015). Delivery of cardiolipins to the Salmonella outer membrane is necessary for survival within host tissues and virulence. Cell. Host. Microbe..

[17] Lin Y, Bogdanov M, Tong S, Guan Z, Zheng L (2016). Substrate selectivity of lysophospholipid transporter LplT involved in membrane phospholipid remodeling in *Escherichia coli*. J. Biol. Chem..

[18] Makula RA, Finnerty WR (1970). Microbial assimilation of hydrocarbons: identification of phospholipids. J. Bacteriol..

[19] Makula RA, Finnerty WR (1972). Microbial assimilation of hydrocarbons: cellular distribution of fatty acids. J. Bacteriol..

[20] Makula RA, Lockwood PJ, Finnerty WR (1975). Comparative analysis of the lipids of Acinetobacter species grown on hexadecane. J. Bacteriol..

[21] Scott CC, Makula SR, Finnerty WR (1976). Isolation and characterization of membranes from a hydrocarbon-oxidizing *Acinetobacter sp*. J. Bacteriol..

[22] Scott CC, Finnerty WR (1976). Characterization of intracytoplasmic hydrocarbon inclusions from the hydrocarbon-oxidizing Acinetobacter species HO1-N. J. Bacteriol..

[23] Torregrossa RE, Makula RA, Finnerty WR (1977). Characterization of lysocardiolipin from *Acinetobacter sp*. HO1-N. J. Bacteriol..

[24] Makula RA, Torregrossa RE, Isle HB (1978). Identification and synthesis of acyl-phosphatidylglycerol in *Acinetobacter sp*. HO1-N. J. Bacteriol..

[25] Angelini R (2012). Lipidomics of intact mitochondria by MALDI-TOF/MS. J. Lipid. Res..

[26] Ren M, Phoon CKL, Schlame M (2014). Metabolism and function of mitochondrial cardiolipin. Prog. Lipid. Res..

[27] Schlame M, Ren M, Xu Y, Greenberg ML, Haller I (2005). Molecular symmetry in mitochondrial cardiolipins. Chem. Phys. Lipids..

[28] Garrett TA, Kordestani R, Raetz CRH (2007). Quantification of cardiolipin by liquid chromatography-electrospray ionization mass spectrometry. Methods. Enzymol.

[29] Arias-Cartin R, Grimaldi S, Arnoux P, Guigliarelli B, Magalon A (2012). Cardiolipin binding in bacterial respiratory complexes: structural and functional implications. Biochim. Biophys. Acta..

[30] Kimura T, Jennings W, Epand RM (2016). Roles of specific lipid species in the cell and their molecular mechanism. Prog. Lipid. Res..

[31] Senff LM, Wegener WS, Brooks GF, Finnerty WR, Makula RA (1976). Phospholipid composition and phospholipase A activity of Neisseria gonorrhoeae. J. Bacteriol..

[32] Mileykovskaya E (2009). Phosphatidic acid and *N*-Acylphosphatidylethanolamine form membrane domains in *Escherichia coli* mutant lacking cardiolipin and phosphatidylglycerol. J. Biol. Chem..

[33] Catucci L, Depalo N, Lattanzio VMT, Agostiano A, Corcelli A (2004). Neosynthesis of cardiolipin in *Rhodobacter sphaeroides* under osmotic stress. Biochemistry (Mosc).

[34] Lobasso S, Palese LL, Angelini R, Corcelli A (2013). Relationship between cardiolipin metabolism and oxygen availability in *Bacillus subtilis*. FEBS Open Bio.

[35] Mileykovskaya E, Dowhan W (2009). Cardiolipin membrane domains in prokaryotes and eukaryotes. Biochim. Biophys. Acta..

[36] Rennera LD, Weibel DB (2011). Cardiolipin microdomains localize to negatively curved regions of *Escherichia coli* membranes. Proc. Natl. Acad. Sci. USA.

[37] Barák I, Muchová K (2013). The role of lipid domains in bacterial cell processes. Int. J. Mol. Sci..

[38] Haines TH, Dencher NA (2002). Cardiolipin: a proton trap for oxidative phosphorylation. FEBS Letters..

[39] Romantsov T, Stalker L, Culham DE, Wood JM (2008). Cardiolipin controls the osmotic stress response and the subcellular location of transporter ProP in *Escherichia coli*. J. Biol. Chem..

[40] Romantsov T, Guan Z, Wood JM (2009). Cardiolipin and the osmotic stress responses of bacteria. Biochim. Biophys. Acta..

[41] Gold VA (2010). The action of cardiolipin on the bacterial translocon. Proc. Natl. Acad. Sci. USA.

[42] Tsatskis Y (2005). The osmotic activation of transporter ProP is tuned by both its C-terminal coiled-coil and osmotically induced changes in phospholipid composition. J. Biol. Chem..

[43] Sutterlin HA, Zhang S, Silhavy TJ (2014). Accumulation of phosphatidic acid increases vancomycin resistance in. Escherichia coli. J. Bacteriol..

[44] Bishop RE (2014). Emerging roles for anionic non-bilayer phospholipids in fortifying the outer membrane permeability barrier. J. Bacteriol..

[45] Maifiah MH (2016). Global metabolic analyses identify key differences in metabolite levels between polymyxin-susceptible and polymyxin-resistant *Acinetobacter baumannii*. Sci. Rep..

[46] Dong H (2016). Structural insights into cardiolipin transfer from the Inner membrane to the outer membrane by PbgA in Gram-negative bacteria. Sci. Rep..

[47] Bertani G (1951). Studies on lysogenesis. I. The mode of phage liberation by lysogenic. Escherichia coli. J. Bacteriol..

[48] Kates, M. Techniques of lipidology, In *Laboratory Techniques in Biochemistry and Molecular Biology* (ed. Burdon, R. H. & Knippenberg, P. H.) 100–110 (Elsevier, 1986).

[49] Sun G (2008). Matrix-assisted laser desorption/ionization time-of-flight mass spectrometric analysis of cellular glycerophospholipids enabled by multiplexed solvent dependent analyte-matrix interactions. Anal. Chem..

[50] Fuchs B, Schober C, Richter G, Süss R, Schiller J (2007). MALDI-TOF MS of phosphatidylethanolamines: different adducts cause different post source decay (PSD) fragment ion spectra. J. Biochem. Biophys. Methods..

