# Supplementary material for: Identification of unique cardiolipin and monolysocardiolipin species in Acinetobacter baumannii
Source: Sci Rep. 2017 Jun 7;7:2972. doi: 10.1038/s41598-017-03214-w (PMC5462836; doi:10.1038/s41598-017-03214-w)
Supplement: Supplementary file 1 — Figure S1 [file 41598_2017_3214_MOESM1_ESM.pdf]

Identification of unique cardiolipin and monolysocardiolipin species in *Acinetobacter baumannii*.

Patrizia Lopalco[1], Julia Stahl[2], Cosimo Annese[3], Beate Averhoff [2], Angela Corcelli[1,4*]

[1]Department of Basic Medical Sciences, Neurosciences and Sensory Organs, University of Bari A Moro, Bari, Italy;

[2]Department of Molecular Microbiology & Bioenergetics, Institute of Molecular Biosciences, Goethe-University Frankfurt am Main, Germany;

[3]CNR-ICCOM, Bari, Italy

[4]Institute for Chemical-Physical Processes, National Research Council (CNR- IPCF), Bari, Italy;

*Correspondence to: Angela Corcelli, Dipartimento di Scienze Mediche di Base, Neuroscienze e Organi di Senso, Università di Bari A. Moro, Piazza G. Cesare, 70124 Bari, Italy. Tel.: + 39 080 5448530. E-mail address: a.corcelli@uniba.it

Supplementary information

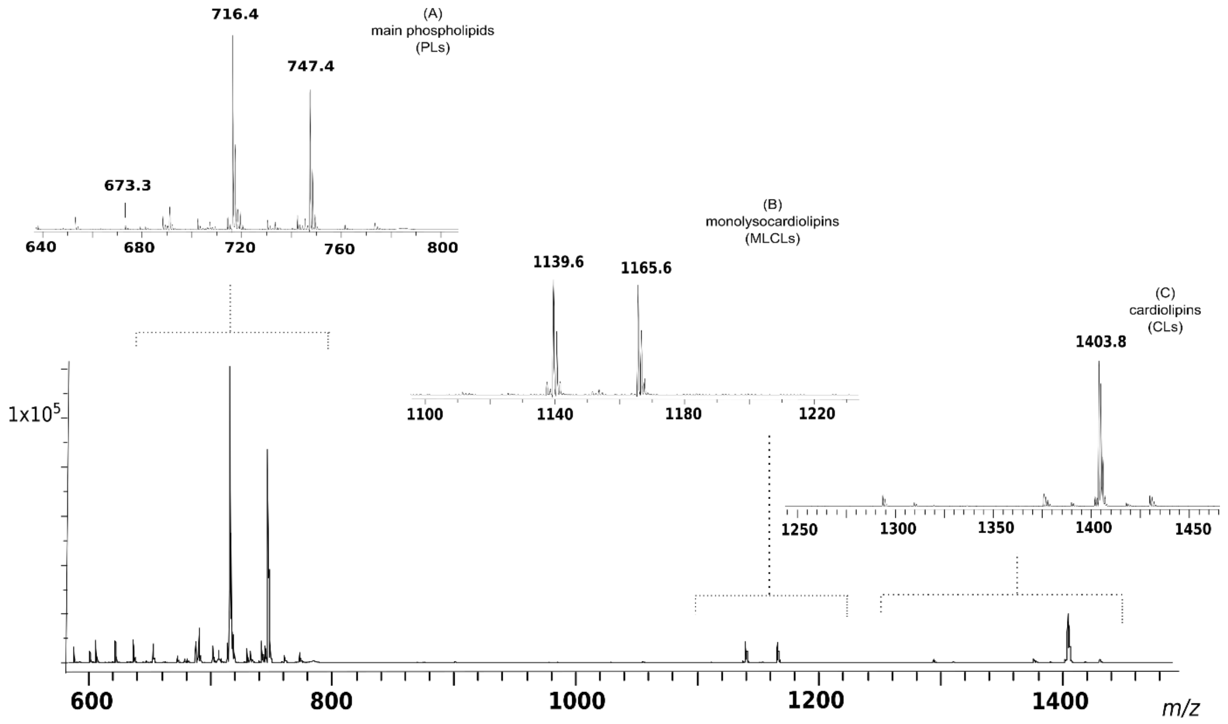

Figure S1. MALDI-TOF/MS lipid profile of intact bacterial membranes of *A. baumannii* (full m/z range). Mass spectra were acquired in negative ion mode using 9-aminoacridine as matrix. Bacterial membrane lipids have been analysed in intact membranes by avoiding lipid extraction and TLC separation steps, by following a procedure previously described in the literature that highly reduces the times of analyses and the possibility of introducing artifacts [25]. Mass spectrum in the lower panel represents the MALDI-TOF/MS lipid profile in the full *m/z* range 600-1500. In panel (A) x-axis enlargement of the *m/z* range 640-800 referable to the main phospholipids (PLs); in panel (B) x-axis enlargement of the *m/z* range 1100-1200 referable to the monolysocardiolipins (MLCLs); in panel (C) x-axis enlargement of the *m/z* range 1350-1450 referable to the cardiolipins (CLs). Peaks detected in intact membranes are the same present in the lipid extract.
